# The rat's not for turning: Dissociating the psychological components of cognitive inflexibility^☆^

**DOI:** 10.1016/j.neubiorev.2015.06.015

**Published:** 2015-09

**Authors:** Simon R.O. Nilsson, Johan Alsiö, Elizabeth M. Somerville, Peter G. Clifton

**Affiliations:** a Department of Psychology, University of Cambridge, Cambridge CB2 3EB, UK; b MRC and Wellcome Trust Behavioural and Clinical Neuroscience Institute, University of Cambridge, Cambridge CB2 3EB, UK.; c School of Life Sciences, University of Sussex, Brighton BN1 9QG, UK; d School of Psychology, University of Sussex, Brighton BN1 9QH, UK; e Department of Neuroscience, Unit of Functional Neurobiology, University of Uppsala, SE-75124 Uppsala, Sweden

**Keywords:** Reversal learning, Attentional set shifting, Discrimination learning, Cognitive flexibility, Animal models

## Abstract

Executive function is commonly assessed by assays of cognitive flexibility such as reversal learning and attentional set-shifting. Disrupted performance in these assays, apparent in many neuropsychiatric disorders, is frequently interpreted as inability to overcome prior associations with reward.

However, non-rewarded or irrelevant associations may be of considerable importance in both discrimination learning and cognitive flexibility. Non-rewarded associations can have greater influence on choice behaviour than rewarded associations in discrimination learning. Pathology-related deficits in cognitive flexibility can produce selective disruptions to both the processing of irrelevant associations and associations with reward. Genetic and pharmacological animal models demonstrate that modulation of reversal learning may result from alterations in either rewarded or non-rewarded associations.

Successful performance in assays of cognitive flexibility can therefore depend on a combination of rewarded, non-rewarded, and irrelevant associations derived from previous learning, accounting for some inconsistencies observed in the literature. Taking this combination into account may increase the validity of animal models and may also reveal pathology-specific differences in problem solving and executive function.

## Highlights

• Non-rewarded or irrelevant prior associations are important for flexible responding.
• Associations of reward and non-reward in reversal learning are neurally dissociable.
• Disruption of prior irrelevant or rewarded associations cause pathological deficits.
• Experimental paradigms of cognitive flexibility can be improved to aid translation.

## 1 Introduction

Successful goal-directed behaviour requires that discrimination learning builds on stimulus-reward associations that are flexible in response to changing situational demands. This capacity is often referred to as ‘cognitive flexibility’. Disruptions in cognitive flexibility are a common feature of neuropsychiatric disorders that are not alleviated by available drugs (Barch, 2005). The extent of cognitive disruption correlates with long-term health outcome (Green, 2006) and the inability to treat these deficits can act as a rate-limiter of treatment progression, trapping patients within life-long dependency (Geyer and Tamminga, 2004).

Impairments in tasks of cognitive flexibility have been associated with diverse pathologies such as Parkinson's disease, autism, Alzheimer's disease, depression, Huntington's disease, ADHD, and schizophrenia. Table 1 serves to highlight both the broad range of disorders linked to cognitive inflexibility and the failure of extant tasks and paradigms to distinguish between these pathologies: patients with widely disparate diagnoses and symptom profiles often display ‘similar’ deficits. For some neuropsychiatric and neurological disorders, this common behavioural marker could represent similar neurobiological dysfunctions produced by variable aetiologies. Neuropsychological markers of disorders that unite clinical diagnoses (endophenotypes) have previously been reported and have received increased focus through the National Institute of Mental Health's Research Domain Criteria Project (Insel et al., 2010); examples include enhanced ‘model-free’ habit learning in obsessive–compulsive disorder, drug addiction and binge eating disorder (Worbe et al., 2015), or increased impulsivity in drug addiction and attention-deficit hyperactivity disorder (Dalley et al., 2011). However, it is also conceivable that currently prevalent reversal and set-shifting paradigms lack sensitivity to detect dissociable impairments manifested as cognitive inflexibility. A more detailed understanding of the cognitive mechanisms that underpin performance on such tests may thus lead to more sensitive assays that reveal pathology-specific differences and, in turn, to rational research strategies that permit the development of more effective therapeutic interventions. Such knowledge could furthermore promote the identification of endophenotypes where they exist and inform both neuropsychiatric research and investigation into the genetic basis of reversal learning and attentional set shifting (Ouden et al., 2013).

Here, commonly used assays of cognitive flexibility are reviewed with a particular focus on reversal learning. We define the underlying components of cognitive flexibility, and stress that learning theory can interpret reversal learning as concurrent schedules that may reveal both *perseverance* and *learned non-reward*. We review experiments designed to separately assess these mechanisms, and discuss their consequences for interpreting reversal learning performance. Although clinical and preclinical assays have begun to define the anatomical loci and neurochemistry involved in cognitive flexibility, the protocols used to assess flexible responding are varied and this may account for some inconsistency in the literature. We outline some potential benefits of resolving reversal learning into tests of its constituent cognitive components, including the possibility of obtaining clearer indications of pathology-specific differences in problem solving and increased validity of their respective animal models.

## 2 Measuring cognitive flexibility

Cognitive flexibility is most commonly assessed in reversal learning and attentional set-shifting tasks. Reversal learning can often involve a single perceptual dimension typically containing two different conditioned stimuli (CS's). Responses to one stimulus are rewarded (CS+) while responses to the second stimulus are non-rewarded (CS−). After learning an initial CS+ versus CS− discrimination, the contingencies are reversed. Attentional set-shifting, in contrast, involves at least two superimposed perceptual dimensions, each containing at least two different stimuli. In an initial acquisition phase of attentional set-shifting, two stimuli within one perceptual dimension serve as CS+ and CS− while stimuli within other dimensions are irrelevant. In the following set-shifting phase, the previous CS+ and CS− become irrelevant while stimuli within a previously irrelevant dimension become the relevant CS+ and CS−.

Reversal learning and attentional set-shifting have been incorporated into the intradimensional/extradimensional task (ID/ED-task) of the Cambridge Neuropsychological Test Automated Battery (CANTAB) which has been used to assess primates and human participants (Dias et al., 1996, Leeson et al., 2009). This task consists of a series of components of increasing level of difficulty. Subjects initially learn a two-choice discrimination where responses to one stimulus are perfectly correlated with reward and responses to the second stimulus are perfectly correlated with the absence of reward. This is followed by a compound discrimination, where a second superimposed sensory or cognitive dimension is introduced but the correct and incorrect stimuli remain constant. Next, subjects are faced with an ID-shift where learned stimuli are replaced by novel stimuli, again with the relevant and irrelevant dimensions remaining constant. Once this discrimination has been acquired, subjects are challenged by an ED-shift, where the irrelevant dimension becomes relevant and the relevant dimension becomes irrelevant. Each of these tests is typically followed by a reversal learning test. There are also analogous bowl-digging (McAlonan and Brown, 2003) and operant (Scheggia et al., 2013) versions of this task used with rodents.

Although superficially similar, attentional set-shifting is more cognitively challenging than reversal learning. This was initially attributed to the partial positive reinforcement of a previously rewarded stimulus after the attentional set shift (Buss, 1953); but this hypothesis was falsified by studies demonstrating that attentional set-shifting is more difficult than reversal learning even after controlling for partial reinforcement (Buss, 1956, Harrow and Friedman, 1958, Kendler and D’Amato, 1955). Yet it should be noted that the latter paradigms contain additional confounds, including memory-related constraints, which may severely challenge meaningful comparisons between the reversal learning and attentional set-shifting (Slamecka, 1968). However, the discrepancy in difficulty is most likely produced by the attentional relocation demanded in attentional set-shifting, but not reversal learning, as well as the suppression of associations of non-correlated reinforcement, or learned irrelevance (Bissonette et al., 2008, Dias et al., 1996).

As an assay of cognitive flexibility, reversal learning has been somewhat overshadowed by attentional set-shifting. One reason for this might be the relatively greater difficulty of attentional set-shifting which may increase the likelihood of observing clinically-relevant deficits and their alleviation through treatment. There is nevertheless evidence suggesting that reversal learning may be a more suitable translational model for clinical application. Attentional set-shifting, but not reversal learning, has been linked to verbal ability and intelligence (Laws, 1999). By comparing groups matched for current IQ, Leeson et al. (2009) found that schizophrenic patients do not differ from healthy controls on attentional set-shifting but do show reversal learning impairments in the CANTAB ID/ED-task. Prompting schizophrenic patients to verbalise their decision-making can remediate poor attentional set-shifting performances (Choi and Kurtz, 2009) and when figural stimuli are replaced by verbal stimuli, the performance of schizophrenic patients deteriorates whilst the performance of healthy controls improves (Rossell and David, 1997). Hence, deficits in attentional set-shifting ability may reflect verbal and intelligence deficits in patients while impairments in reversal learning are relatively independent of intelligence (Leeson et al., 2009). Reversal learning is also a better predictor of social functioning (Shamay-Tsoory et al., 2007).

## 3 Behavioural analyses of discrimination learning

Discrimination learning occurs in response to dissociable reinforcements of stimuli, perceptual dimensions or contexts and the nature of subsequent reversal learning and attentional set-shifting processes largely depend on how these discriminations are acquired. Early views held discrimination learning to be a low-level comparative process where behaviour is controlled by relational features of stimuli and rules are acquired through trial-and-error processes. However, this account had difficulty with phenomena such as stimulus generalisation and reversal following transposition tests (Spence, 1937). Spence suggested that discrimination learning depended on both excitation and inhibition (Spence, 1936). Positive reinforcements increase the excitatory strength of a stimulus and elicit approach, while non-reinforcements decrease the excitatory strength of a stimulus and make approach less likely. While Spence treated non-reinforcement as a non-aversive mechanism inhibiting the excitatory strength and approach tendency associated with a stimulus, others have considered non-reinforcement to result in aversive negative reinforcement (Amsel, 1958, Behar, 1961, Nissen, 1950, Terrace, 1971). However, Spence's conditioned excitatory–inhibition theory predicts that previously non-reinforced stimuli, or stimuli not correlated with reinforcement, will acquire excitatory tendencies at a similar or faster rate than neutral stimuli. The theory therefore fails to explain phenomena such as latent inhibition or learned irrelevance, in which such associations delay learning. The mechanisms of excitation and inhibition are also insufficient to explain phenomena such as the serial-reversal effect (Mackintosh et al., 1968) where later reversals are acquired at a faster rate than earlier reversals, or the overtraining reversal effect, where prolonged discrimination training can facilitate reversal learning (Lovejoy, 1966).

To account for these phenomena, it has been necessary to involve the additional mechanisms of attentional stimulus selection-processes and predictability (Mackintosh, 1983, Pearce and Hall, 1980). Attention is a composite term for processes ensuring appropriate and continued maintenance and selection of stimuli for goal-directed behaviour (Parasuraman, 1998). In the context of discrimination learning and cognitive flexibility, attention is thought of as a determinant of perception allowing stimuli predictive of reinforcement to gain excitatory or inhibitory conditioning while irrelevant information fail to interfere with these processes (Mackintosh, 1965). In attentional theory, it is suggested that subjects attend to those stimuli (Mackintosh, 1975), or stimulus dimensions (Sutherland and Mackintosh, 1971), that are the best predictors of the reward contingencies, and this attentional allocation subsequently drives responding towards the appropriate CS+ and CS−. This approach suggests that although all forms of discrimination learning and cognitive flexibility require attentional *allocation*, only extradimensional set-shifting require attentional *relocation* and will therefore be acquired at a slower rate. Overtraining reversal effects (Mackintosh, 1969) and serial reversal effects (Mackintosh et al., 1968) may also be explained by extensive training increasing attentional *allocation* to particular discriminative features.

In sum, although theoretical approaches to animal learning differ in the value they place on attentional factors and reinforcement contingencies throughout the process of discrimination acquisition, they explain differential responding to distinctive stimuli in terms of independent mechanisms of excitation versus inhibition and consequent changes in attention to the relevant stimuli.

## 4 Dissociating the components of cognitive flexibility

The implication of this analysis is that pathology-related cognitive inflexibility, revealed by disturbances in reversal learning or attentional set shifting, may be due to abnormalities in one or more separate processes. In reversal learning, the initial two-choice discrimination can be resolved to an excitatory CS – US association, eliciting approach and contact, and an inhibitory CS – ‘no US’ association, eliciting avoidance. After the subsequent contingency shift, the CS predicting the US becomes associated with ‘no US’, a process opposed by perseverance. Conversely, the CS initially predicting ‘no US’ now predicts the US, a process opposed by learned non-reward.

Similarly, in attentional set-shifting, the initial discrimination is likely to depend on conditioned attention towards the relevant dimension and conditioned inattention towards the irrelevant dimension. After the subsequent contingency shift, the relevant dimension becomes irrelevant, a process opposed by perseverance. Conversely, the irrelevant dimension becomes relevant, a process opposed by learned irrelevance. Deficits in reversal learning and attentional set-shifting can therefore be interpreted as failure to dissipate prior associations of previous positive (*perseverance*) and negative (*learned non-reward* or *learned irrelevance*) outcome together with any associated attentional effects.

### 4.1 Perseverance

‘Perseveration’ describes a range of phenomena related to the inappropriate repetition or maintenance of an activity or an abstract rule (Crider, 1997). This includes, for example, various forms of catatonia-like repetitions of motor-outputs (Freeman and Gathercole, 1966, Helmick and Berg, 1976, Luria, 1965, Sandson and Albert, 1984). In cognitive terms perseveration is most often used to describe an executive functioning deficit causing the repetition or maintenance of abstract information encoding for relationships between stimuli and goals (Garner, 2006). Perseveration could potentially also be affected by episodic retrieval mechanisms of task rules (Frank and Fossella, 2010) and motor impulsivity (Izquierdo and Jentsch, 2012). However, there is little to suggest that these mechanisms would have separable effects on discrimination learning, perseveration and learned non-reward or learned irrelevance in assays of cognitive flexibility. It is notable that the converse of cognitive flexibility – habitual responding – is ubiquitous in animal behaviour and has always been interpreted as critical for allowing efficient and rapid responding to environmental challenges as well as a way of freeing cognitive resource (James, 1890). However, excessive perseverance has also long been recognised as a component of psychopathology (Bleuler, 1950).

Perseveration need not be delimited by the outcome of the association that is inappropriately repeated or maintained. Thus excessive avoidance of a previous CS− and excessive approach of a previous CS+ are both potentially examples of perseveration. Yet discussions of perseveration are typically phrased in terms of an inability to overcome reinforced, rather than irrelevant or non-reinforced associations. The majority of reported manipulations affecting cognitive flexibility are also interpreted as due to effects involving reinforced associations or perseverance (Abdul-Monim et al., 2007, Boulougouris et al., 2008, Floresco et al., 2006).

### 4.2 Learned non-reward

Learned non-reward is the consequence of the CS—‘no US’ association formed in a two-choice discrimination paradigm. After a contingency shift, learned non-reward is expressed as interference with learning of a new CS–US association after prior experience of non-reward with that CS. Recent use of the term originates from a study describing the role of the orbitofrontal cortex (OFC) in reversal learning (Tait and Brown, 2007) although similar descriptors were used earlier in this context (e.g., learned avoidance of non-reward) (Behar, 1961). It is closely related to the phenomenon of latent inhibition. The difference between latent inhibition and learned non-reward is most marked in simultaneous discrimination and reversal paradigms where the CS+ and CS− are presented concurrently. In successive reversal tasks, such as go/no-go paradigms, the two stimuli are presented independently. In these tasks the presentation of a previously non-rewarded but now rewarded CS can therefore be nearly identical to an appetitive assay of latent inhibition.

The inability to overcome a non-rewarded association in two-choice reversal learning has also been referred to as learned avoidance (Clarke et al., 2007) and learned irrelevance (Boulougouris et al., 2008). However, none of these terms accurately capture the phenomena occurring in appetitive reversal learning (Table 2). Learned non-reward is produced when a stimulus now positively correlated with reward has been previously negatively correlated with reward. Learned avoidance is produced when a now neutral stimulus has been previously correlated with aversive reinforcement. Learned irrelevance is produced when a stimulus now positively correlated with reward has been previously non-correlated with reward.

### 4.3 Errors as measures of performance

The most common approach for assessing perseverative responding in reversal learning is to classify incorrect responses into one of two categories; either as an ‘early-error’ or ‘perseverative error’, or as a ‘late-error’ or ‘regressive error’. For example, in operant tasks, early or perseverative errors have been indexed by repetitive bouts of responding towards the previous CS+ (Boulougouris and Robbins, 2010) or incorrect responses made early in reversal when the majority of responses are to the previous CS+ (Boulougouris et al., 2008). In maze tasks incorrect responses made before making a first response to the new CS+ (McCool et al., 2008), or three such responses in a block of four trials (Dias and Aggleton, 2000, Palencia and Ragozzino, 2004), have been used, depending on the experimental protocol. Other incorrect responses are then classified as late errors.

It is often assumed that the number of early errors reflects the stability of the original CS–US association, or perseverance, while late errors are considered to reflect general cognitive abilities related to attention and the acquisition of an alternative CS–US association. However, in experiments in which early errors and late errors are analysed, previously correct and incorrect CSs are presented during all reversal trials. Previous excitatory and inhibitory conditioning can therefore influence choice-behaviour in both early and late phases of learning. It may also be that the presence of a CS− invigorates the rate of responding to a CS+ (Mackintosh, 1974, Terrace, 1963). Analyses of early errors and late errors may nevertheless be valuable in other ways. Early and late errors may potentially reflect failures in memory processing. For example, Palencia and Ragozzino (2004) discuss, and then reject, the possibility that an increase in regressive, late errors resulting from blockade of dorsomedial striatal NMDA receptors in a place reversal task might have arisen from a disruption of working memory that impaired the ability to recall earlier non-reinforced trials. More generally, similar patterns of error patterns during early and late phases of learning in two different reversal tasks may indicate that the two tasks are solved using similar approaches or that they depend on similar underlying neural mechanisms (Boulougouris et al., 2007, Chudasama and Robbins, 2003a).

### 4.4 Preclinical assays of perseverance and learned non-reward

Ever since learning theorists stressed that discrimination learning is a two-process phenomenon (Amsel, 1958, Behar, 1961, Nissen, 1950, Spence, 1936), experimental efforts have been made to determine the relative contribution of reinforcement and non-reinforcement in discrimination and reversal learning. The paradigms used in these studies include measures of reversal learning as well as perseverance and learned non-reward and therefore provide superior ‘resolution’ relative to standard discrimination and reversal protocols. Yet results have varied across protocols and species, with data indicating that both reinforced and non-reinforced contingencies can guide responding.

One approach has been to train subjects on the contingency of a single stimulus prior to either a discrimination or reversal challenge (Table 3). CS+ pre-exposure should facilitate performance relative to CS− pre-exposure if responses during reversal learning or discrimination learning are primarily guided by reward. Conversely, CS− pre-exposure should facilitate performance relative to CS+ pre-exposure if responses during reversal learning primarily are guided by non-reward. In these protocols, performances have indeed been found to be best if the subject has been pre-exposed to the CS− prior to discrimination learning (Blomquist et al., 1973, Moss and Harlow, 1947) or been pre-exposed to non-rewarded responses to the previous CS+ prior to reversal learning (Cross and Brown, 1965, Sasaki, 1969). This suggests that associations with non-reward have a greater role in discrimination and reversal learning than associations with reward. Nevertheless, it has been suggested that the superior performance from CS− pre-exposure in studies of discrimination learning may be due to an attraction to novelty (Grabbe and Campione, 1969). Others have also found that pre-reversal CS+ learning has a bigger effect on reversal learning (Vaughter and Cross, 1965). These inconsistencies are likely to be related to the use of different species, different stimuli dimensions, and the number of single-stimulus pre-exposure trials.

The importance of the CS+ and the CS− in discrimination and reversal learning can also be assessed by replacing or introducing variability in either the CS+ or CS− (Table 4). When the CS+ is replaced or varied, the only reliable predictor of reinforcement across trials is the CS−. When the CS− is replaced or varied, the only reliable predictor of reinforcement across trials is the CS+. Stimulus variability has been introduced during discrimination learning to assess the relative roles of reinforcement and non-reinforcement on discrimination learning, while others have included a contingency shift prior to stimulus replacement to assess the role of perseverance or learned non-reward in reversal learning. Within these paradigms, subjects have often been observed to make more errors with CS− variability than CS+ variability (Goulart et al., 2005, Mullins and Winefield, 1979, Stevens and Fechter, 1968) indicating that that successful performance in discrimination and reversal learning primarily dependent upon avoidance of the CS−. However, reports have not always been consistent, with some protocols showing that CS+ variability can retard performance more than CS− variability (Gardner and Coate, 1965).

Of further note is a 3-choice simultaneous visual reversal task (Jentsch et al., 2002) that has been suggested to give a measure of discernment of stimulus perseveration (Table 5). Here, vervet monkeys were presented with an initial discrimination where one stimulus was designated as CS+ while two other stimuli were designated as CS−’s. Following reversal, the previous CS+ became a CS−, and one of the previous CS−’s became the new CS+. The third stimulus remained non-rewarded during both discrimination and reversal learning. Responses to the previously rewarded but now non-rewarded stimulus were coded as perseverative while responses to the consistently non-rewarded stimulus gave a control for random responses that may be expected during a search for an alternative response strategy, or as result of a more general invigoration of responding. However, in this paradigm, all three stimuli (stimuli that were previously non-rewarded but are now rewarded, stimuli previously rewarded but now non-rewarded, and stimuli remaining non-rewarded) are presented in both the initial discrimination and subsequent reversal trials. This may make interpretations of stimulus avoidance versus approach strategies difficult.

In sum, although interpretations of preclinical data often build on the assumption that reinforced associations guide choice behaviour in reversal paradigms, the above experiments serve to highlight that non-reinforcement also can be of considerable importance.

### 4.5 Learned irrelevance and perseverance in attentional set-shifting tasks

Learned irrelevance in an attentional set-shifting task is the analogue of learned non-reward. In learned irrelevance, a stimulus dimension initially non-correlated with reinforcement becomes correlated with reinforcement. That is, a stimulus dimension previously rewarded 50% of the time becomes rewarded 100% of the time (Table 2).

Learned irrelevance is more difficult to overcome than latent inhibition and the discrepancy in difficulty between reversal learning and attentional set-shifting may be related to the discrepancy in difficulty between learned non-reward and learned irrelevance (Buss, 1956, Kendler and D’Amato, 1955). Learned irrelevance could be viewed as the product of the simpler mechanisms of pre-exposure to the CS as well as the US, or as representing the combined effect of latent inhibition and US pre-exposure (Rescorla and Holland, 1982). However, later data has favoured learned irrelevance as a non-reducible independent phenomenon (Bennett et al., 1995).

In contrast to the dissociation between learned non-reward and perseverance, the dissociation of learned irrelevance and perseverance in attentional set-shifting has been investigated in clinical populations. The approach has been to modify the CANTAB ID/ED-task. Either the previously relevant dimension is replaced by a novel irrelevant dimension to probe learned irrelevance, or the previously irrelevant dimension is replaced by a novel relevant dimension to probe perseverance (Table 6). Using this method in a non-clinical human group, learned irrelevance has been shown to contribute more than perseverance to the difficulty of attentional acquisition (Maes and Eling, 2009) as well as attentional set-shifting (Maes et al., 2004) paralleling the often more prominent role of learned non-reward in preclinical tests of reversal learning. These studies have also revealed pathology-specific dissociations. Humans with prefrontal damage (Owen et al., 1993), individuals with schizophrenia (Elliott et al., 1995, Elliott et al., 1998) or Huntington's disease (Lawrence et al., 1999) all exhibit perseverative set-shifting deficits, while non-medicated individuals with Parkinson's disease show deficits in both perseverance and learned irrelevance (Owen et al., 1993). However, l-Dopa medication in Parkinson's disease is associated with selective impairments in overcoming learned irrelevance (Owen et al., 1993, Slabosz et al., 2006). This suggests that perseverance, but not learned irrelevance, is related to dopaminergic hypoactivity. In the rodent, this approach to measuring attentional set-shifting has only been reported once, with an assessment of perseverance, but not learned irrelevance, in mice using the bowl-digging task (Garner et al., 2006).

### 4.6 Non-responding in discrimination and reversal tasks

In discrimination and reversal tasks, failures to respond or omissions are often included and discussed as controls for motivational or motoric confounds. Yet in discrimination learning theory, non-responding towards the CS− in a simultaneous discrimination is variously interpreted as a product of the CS− lacking excitatory strength (Spence, 1936), being aversive or acting as a conditioned inhibitor (Amsel, 1958, Behar, 1961, Nissen, 1950, Terrace, 1971), having acquired inattention (Sutherland and Mackintosh, 1971), or resulting from a lack of attention, due to being an accurate predictor of non-reward (Pearce and Hall, 1980). In addition to measuring motivation or motoric factors, non-responding in discrimination and reversal assays could therefore also be seen as a direct consequence of learning, most readily produced by associations with non-reward.

After initial high rates of responding towards the previous CS+ immediately following a contingency shift, the excitatory strength or attentional control of the CS+ decreases and the excitatory strength of both CS+ and CS− should be similarly low. Thus, the previous CS+ becomes associated with non-reward in early reversal learning while the previous CS− remains associated with non-reward from conditioning in discrimination learning and this can cause a high number of non-responses or omissions.

However, some experiments suggest that omissions in reversal learning can also be used as a measure of learning. For example, in serial operant reversal tasks, omissions may show a serial reversal effect and decrease across subsequent reversal tests (Boulougouris et al., 2008, Nilsson et al., 2012) indicating that non-responding is a measure of learning as well as of motivation and motoric factors. Also, in a rat bowl-digging paradigm (Tait and Brown, 2007) and a mouse operant paradigm (Nilsson et al., 2012), omissions are more prominent in a learned non-reward test, where the previous CS− is paired with a novel CS, than in a perseverance test, where the previous CS+ is paired with a novel CS, indicating that omissions in tasks of reversal learning can be specifically related to learning and learned non-reward.

Notably, response omissions are also a primary measure of learning in other tasks assessing cognitive flexibility, including successive reversal learning paradigms (Burke et al., 2009, McEnaney and Butter, 1969, Nonkes et al., 2011, Schoenbaum et al., 2003) and latent inhibition (Lubow, 1989). Thus omissions should be considered relevant to learning in simultaneous discrimination and reversal tasks and not simply discussed as potential controls for motor or motivational side effects of an experimental manipulation.

### 4.7 Novelty confounds

A novel stimulus or a novel stimulus dimension is present in most studies that feature separate tests of perseverance and learned non-reward or learned irrelevance in simultaneous discrimination procedures. Changes in novelty attraction or avoidance could therefore confound any interpretation regarding the effect of an experimental manipulation on learning. In a perseverance test, a novel rewarded stimulus or dimension is paired with a previously rewarded but now non-rewarded stimulus or dimension. In this test increased novelty attraction could be misinterpreted as facilitated learning while increased novelty avoidance could be misinterpreted as retarded learning. In a learned non-reward or learned irrelevance test, a novel non-rewarded stimulus or dimension is paired with a previously non-rewarded but now rewarded stimulus or dimension. In this test increased novelty attraction would be observed as retarded learning while increased novelty avoidance would be observed as facilitated learning.

One intrinsic control for a manipulation of novelty attraction or avoidance in these tasks is that it would cause opposing effects on measures of learning in the perseverance and learned non-reward or learned irrelevance tests (Clarke et al., 2007). For example, a manipulation-induced or pathology-related increase in novelty attraction would improve performance in a perseverance test, in which the novel CS is correct, but retard performance in a learned non-reward test, in which the novel CS is incorrect.

Performance in a reversal learning test could also control for effects on novelty attraction or novelty recognition as no novel stimuli are presented in this test. If an effect of a manipulation is observed in perseverance and/or learned non-reward tests where novelty is a feature, as well as in a reversal learning test which lacks novelty, a plausible interpretation would be that the effects are related to shared features of the tests and unrelated to test differences in the presentation of a novel stimulus.

A further approach to account for novelty-related effects is to add control tests of discrimination learning where increases in perseverance and learned irrelevance would facilitate learning. In a perseverance control condition, this can be done by replacing the CS− with a novel CS− while the CS+ remains constant. In a learned non-reward control condition, the CS+ is replaced by a novel CS+, while the CS− remains constant (Gauntlett-Gilbert et al., 1999). Notably, these discrimination tests are identical to the tests designed to assess the role of negative and positive associations in discrimination learning described above (Goulart et al., 2005, Mullins and Winefield, 1979, Stevens and Fechter, 1968). Importantly, this experimental configuration still allows for a novelty confound, as it involves a choice between a previously relevant or irrelevant dimension and a novel dimension (Slabosz et al., 2006). This form of novelty control has as yet only been performed in the context of attentional set-shifting (Gauntlett-Gilbert et al., 1999) but could also be useful in reversal learning.

Novelty confounds in tests of perseverance and learned non-reward can also be investigated using independent tests of spontaneous recognition memory (Nilsson et al., 2013). These tests are non-reinforced and novelty preference is studied in the absence of interference from reinforcement learning. As no overtly reinforced learning is involved, previous conditioning does not affect responses to novelty. Such tests consist of a sample-phase and a test-phase. In the sample-phase, the two stimuli featuring in discrimination learning are presented and approaches are measured. In the test-phase, one of the two stimuli is replaced by a novel stimulus and novelty preference is measured by the ratio of approach to the novel stimulus relative to the total approaches to both stimuli.

We recently developed an additional approach to investigate learned non-reward and stimulus-perseveration in a two-choice visual discrimination and reversal learning task for rats, by interleaving the visual discrimination (CS+ vs. CS−) trials with trials in which either the CS+ or the CS− was paired to a third, neutral stimulus (Table 8; Alsiö, Mar, Nilsson, and Robbins, unpublished). Responses to the CS+ are rewarded, responses to the CS− are non-rewarded, and responses to the third stimulus (CS_50/50_) during the probe trials are rewarded on 50% of the trials. Animals thus learn to choose the CS_50/50_ over the CS−, and to choose the CS+ when this stimulus is presented with the CS_50/50_; each of these pairs is presented once every 10 trials, with the remaining 8 trials being standard CS+ vs. CS−.

**Fig. 1:**
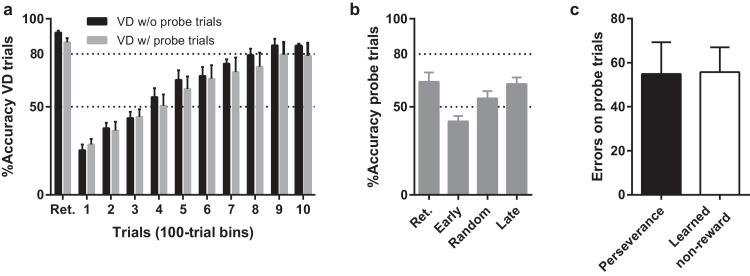
Performance of Lister Hooded rats on a touchscreen reversal learning task with interleaved CS+ vs. CS− presentations (visual discrimination, VD) and probe trials investigating perseverance and learned non-reward. Retention (Ret.) represents performance on the last 100 trials preceding reversal. ‘Early’ represents the first 100 trials after reversal, ‘Random’ represents 100 trials when performance on the VD trials have reached 50%, and ‘Late’ represents 100 trials when the rats have reached 80% accuracy on the VD trials. (a) Reversal-learning performance in the probe task does not differ from control rats tested on the VD trials only (test group: *F*_1,10_ = .046, *p* = .834, group × group *p* = *F*_9,90_ = .714, *p* = .695). (b) Accuracy on probe trials, i.e. choosing CS50/50 over CS− and choosing CS+ over CS50/50, is significantly above chance (50%) during retention (one-sample t-test, *t*_6_ = 2.703, *p* = .035) and late reversal phases (*t*_6_ = 3.532, *p* = .0123), whereas performance drops below chance during early reversal (*t*_6_ = 2.677, *p* = .0367). (c) Number of errors on perseverance and learned non-reward probe trials does not differ (paired *t*-test, *t*_6_ = 0.242, *p* = .817), indicating that the task equally assesses previous positive and negative associations in reversal learning.

Importantly, in the subsequent reversal stage, the previous CS+ becomes CS− and the previous CS− becomes CS+ while the CS_50/50_ remains unchanged. In trials of full reversal learning, the previous CS− is paired with the previous CS+. The two different probe trials are still presented once each every 10 trials, but now represent learned non-reward (previous CS− vs. CS_50/50_) and perseverance (previous CS+ vs. CS_50/50_), allowing within-subject comparisons of these two processes. This paradigm offers a significant advantage over stimulus-replacement designs, as novelty attraction and novelty avoidance cannot confound data interpretations as no novel stimulus is presented. The data (Fig. 1) indicate that previous positive and negative associations both guide responding in reversal learning, and also suggest that there is individual variability in approach and avoidance behaviour in the rat, which is in agreement with work from discrimination learning using the probabilistic selection task (see below).

## 5 Neural mechanisms of association-dependent responding in two-choice discrimination learning and reversal learning

The neural substrates of discrimination and reversal learning have been the subject of extensive investigation (Ragozzino, 2007). Such studies have highlighted the involvement of prefrontal, especially orbitofrontal, cortex, dorsal striatum and amygdala (Frank and O’Reilly, 2006, Keeler and Robbins, 2011), and of a variety of neurotransmitter systems, including 5-HT (Daw et al., 2002, Roberts, 2011), dopamine (Frank and O’Reilly, 2006), and acetylcholine (Robbins and Roberts, 2007). Moreover, attentional set-shifting and reversal learning has been shown to anatomically dissociable; while attentional set-shifting primarily depend upon activity along the prefrontal medial wall (the primate dorsolateral prefrontal cortex or rodent medial prefrontal cortex), the OFC is required for reversal learning (Keeler and Robbins, 2011, Tait et al., 2014). Electrophysiological recordings and imaging studies have also revealed spatial and neural segregations on responses to punishments and rewards (Kringelbach, 2005, Schultz, 1998, Ursu and Carter, 2005), yet explicit investigation of the neural mechanisms underpinning association-dependent responding in tasks of cognitive flexibility has been relatively rare.

### 5.1 Non-human preclinical approaches

Lesions or inactivation of the OFC retard reversal learning in the rodent (Bissonette et al., 2008, Bussey et al., 1997, Chudasama and Robbins, 2003b, Ghods-Sharifi et al., 2008, Graybeal et al., 2011, Kim and Ragozzino, 2005, McAlonan and Brown, 2003, Schoenbaum et al., 2002). This deficit has been further explored in a bowl-digging paradigm separately probing perseverance and learned non-reward by challenging rats in conditions where either the previously correct or incorrect olfactory or somatosensory stimulus reverses contingency and is paired with a novel stimulus of the opposing contingency. In this paradigm, OFC-lesions have been observed to impair performance in a learned non-reward test, but facilitated performance in a perseverance test (Tait and Brown, 2007). Similar opposing effects on learned non-reward and perseverance has been observed in an egocentric spatial task in mice challenged with the 5-HT_2C_ receptor (5-HT_2C_R) antagonist SB242084 (Nilsson et al., 2013) which has been shown to affect reversal learning through activity in the OFC (Boulougouris and Robbins, 2010). The effect of 5-HT_2C_R antagonism on cognitive flexibility also appears selective to reversal learning, as SB242084 fails to affect attentional set-shifting (Baker et al., 2011).

Furthermore, OFC-specific 5,7-DHT-induced 5-HT depletions impaired visual reversal learning in the marmoset (Clarke et al., 2007). 5-HT depleted animals showed deficits in a perseverance test, where the previous CS+ becomes incorrect and is paired with a novel CS+, but performed as well as controls on a learned non-reward test where the previous CS− became correct and was paired with a novel CS− (Clarke et al., 2007). Also, using an analogous visuospatial operant assay, 5-HT_2C_R KO mice and mice systemically treated with the 5-HT_2C_R antagonist SB242084 showed facilitated reversal learning and decreased learned non-reward, but did not differ from controls in a perseverance test (Nilsson et al., 2012).

A further method to dissociate perseverance and learned non-reward is to use Pavlovian successive reversal tasks. This approach has been used with blocked presentations of either CS+ or CS− followed by reward, which thus potentially gives insight into perseverance and learned non-reward. In this task, presumed OFC-inactivation, with intracerebral infusion of a baclofen/muscimol mixture, impaired reversal learning (Burke et al., 2009). Importantly, OFC-inactivation did not affect the rats’ ability to extinguish responding during the previous CS+, but retarded their ability to start responding during the previous CS−. This suggests that activation of the OFC is required for overcoming learned non-reward rather than perseverance (Burke et al., 2009). Conversely, the 5-HT transporter KO rat showed facilitated performance in a similar, though non-blocked, Pavlovian two-choice auditory reversal task (Nonkes et al., 2011). The mutant strain developed faster responding towards the previous CS− (opposed by learned non-reward) but did not differ from wild-type animals in responding towards the previous CS+ (opposed by perseverance). The improvement therefore appeared to be due to enhanced suppression of learned non-reward rather than increased ability to overcome previous positive associations leading to perseverance.

Although the role of the OFC in reversal learning has recently been questioned (Stalnaker et al., 2015), with particular reference to a reported dissociation of the effects of aspiration and excitotoxic lesions on reversal learning in the macaque (Rudebeck et al., 2013), it is not obvious how the results of the studies using temporary OFC activation or intracerebral administration of serotonergic agents reviewed earlier would be accommodated by this hypothesis.

### 5.2 Human preclinical and clinical approaches

Clinical studies of mechanisms underpinning association-dependent responding in two-choice discrimination and reversal learning have typically used either the unexpected outcome task (Cools, 2006) or the probabilistic stimulus selection (PSS) task (Frank et al., 2004). These tasks are dissimilar to standard preclinical tasks in the use of probabilistic rather than fully predictive cues (the PSS task) and one-trial Pavlovian learning rather than prolonged instrumental learning (the unexpected outcome task). Nevertheless, data derived from these tasks support the idea that pharmacological effects, genetic functions and pathology-related dysfunctions in discrimination and reversal learning can depend on the selective processing of positive or negative outcomes.

Furthermore, data in both paradigms has most often been interpreted the context of striatal dopamine signalling. The positive prediction error represented by phasic dopamine release from fast-spiking ventral tegmental cells may increase activity at the D_1_ receptor (D_1_R) and promote stimulus approach learning, while the negative prediction error represented by decreased tonic dopamine levels attenuate activity at the D_2_R and promote stimulus avoidance learning. It has been argued that the two processes are necessary for cognitive flexibility and sufficient to explain reinforcement and non-reinforcement learning (Frank and O’Reilly, 2006).

The unexpected outcome task taps stimulus avoidance and approach strategies in Pavlovian serial reversal learning (Cools, 2006). Participants are presented with a simultaneous two-choice discrimination with one stimulus highlighted by a black border. After a series of correct responses, a contingency reversal is signalled by an unexpected rewarded trial to the previously incorrect stimulus or unexpected loss of reward to previously rewarded stimuli. The ability to overcome previous positive associations is measured by performance on trials immediately preceding unexpected loss of reward, whereas the ability to overcome previously negative associations is measured by performance on the trials immediately preceding unexpected rewards.

In this task, central 5-HT and dopamine signalling appear to have dissociable effects on learned non-reward and stimulus perseveration. Acute tryptophan depletion causes selective increases in the prediction of loss of reward (Cools et al., 2008) and depressed patients show decreased accuracies and anterior ventrolateral putamen blood oxygen level-dependent (BOLD) response following unexpected reward but normal performances following unexpected loss of reward (Robinson et al., 2012). Unexpected rewards but not unexpected loss of reward are related to increased BOLD activation in the posterior dorsolateral striatum (Robinson et al., 2010a). Conversely, l-Dopa and the D_3_R agonist pramipexole impair learning from unexpected loss of reward but not unexpected rewards in individuals with mild Parkinson's disease The D_2_R agonist bromocriptine improves learning following unexpected loss of reward but not unexpected rewards in individuals with high striatal dopamine synthesis capacity (Cools et al., 2009) and reduced dopamine synthesis through acute tyrosine and phenylalanine depletion can improve learning from unexpected loss of reward (Robinson et al., 2010b). Thus, central 5-HT depletion and depression appear to selectively affect learned non-reward while central dopaminergic manipulations have more prominent effects on perseveration. These data are in general agreement with preclinical findings from constitutive or pharmacological 5-HT manipulations affecting responding to previously incorrect but not previously correct stimuli in rodent reversal learning (Nilsson et al., 2012, Nonkes et al., 2012)) and discussions implicating dopamine signalling in perseverative responding using instrumental reversal learning (Clarke et al., 2011, Clatworthy et al., 2009) and attentional set-shifting tasks (Owen et al., 1993).

In contrast to the unexpected outcome task, the PSS task contrasts response strategies only in discrimination learning by employing transitive inference of six probabilistic reward contingencies acquired in separate two-choice discriminations (Frank et al., 2004, Frank et al., 2007b). As shown in Table 7, participants initially learn three two-choice discriminations (AB, CD, EF). In the AB discrimination, stimulus A is rewarded on 80% of trials while stimulus B is rewarded on 20% of the trials. In the CD and EF discriminations, stimulus C and stimulus E are rewarded 70% and 60% of the trials while stimulus D and stimulus F are rewarded 30% and 40% of the trials, respectively. In subsequent probe tests, stimulus A and B are paired with stimuli C, D, E, and F to contrast stimulus approach learning (‘Choose A’ discriminations: AC, AD, AE, AF) with stimulus avoidance learning (‘Avoid B’ discriminations: BC, BD, BE, BF). If responses primarily are guided by reward, performance should be better in the ‘Choose A’ discrimination as these include the stimulus with the greatest reward predictability. If responses primarily are guided by avoidance, performance should be better in the ‘Avoid B’ discrimination as these include the stimulus with the lowest reward predictability. This is consistent with the suggestion that two processes are required for reversal learning (Frank and O’Reilly, 2006); stimulus approach learning is needed to overcome previous negative associations (learned non-reward) while stimulus avoidance learning is required to overcome previous positive associations (perseverance). Notably, these transitive inference tests also contain shared features complicating performance interpretations. Only probe tests of approach learning consist of discriminations between stimuli of positive probabilistic reward contingencies (discriminations: AC and AE) and only probe tests of avoidance learning consist of discriminations between stimuli of negative probabilistic reward contingencies (discriminations: BF, BE). Nevertheless, both tests of reward learning and avoidance learning consist of discriminations between stimuli of positive and negative reward contingencies (discriminations: AD, AF and BF, BE). Hence general deficits in reinforcement learning would produce decreased accuracy in both the ‘Choose A’ and ‘Avoid B’ and conditions, and it may be erroneous to conclude that this is due to disparate deficits in two processes. General deficits in reinforcement learning may also mask more selective effects in approach versus avoidance learning.

Some studies using this paradigm have reported dopaminergic-related functions in avoidance but not approach learning. This includes the single-nucleotide polymorphisms affecting D_2_R mRNA and DARPP-32 mRNA expression and D_2_R density (Cavanagh et al., 2014, Frank and Hutchison, 2009, Frank et al., 2007a). Medication in Parkinson's disease can also have a greater effect on avoidance than approach learning (Frank et al., 2007b) which would be in accordance with the selective effects of l-dopa on perseverative responding in an attentional set-shifting task using a stimulus replacement design (Owen et al., 1993).

There are several reports of selective genetic, pharmacological and pathological effects on reward relative to avoidance learning. Relative to carriers of the DARPP-32 G allele, DARPP-32^A/A^ carriers show improved reward relative to punishment learning (Frank et al., 2007a, Frank and Hutchison, 2009) and the D_2_R antagonist amisulpride improves (Jocham et al., 2011) while the the D_2_R agonist cabergoline impairs (Frank and O’Reilly, 2006) reward learning without affecting avoidance learning. Similarly, schizophrenic patients show impaired reward learning but intact avoidance learning (Gold et al., 2012, Waltz et al., 2007). This effect may be associated with negative symptom expression (Gold et al., 2012, Waltz et al., 2007), which would be in accordance with the learned non-reward and reversal learning deficits observed in depressed patients using the unexpected outcome task (Cools et al., 2008, Robinson et al., 2012). Moreover, ADHD patients *off* medication show deficits in both avoidance and approach learning while ADHD patients *on* medication show selective deficits in avoidance learning (Frank et al., 2006). This would suggest that stimulants improve approach learning in ADHD patients, while avoidance learning manifested by perseverative responding in tasks of cognitive flexibility (Frank and O’Reilly, 2006) is not addressed by currently available drugs. Lastly, the COMT single-nucleotide polymorphisms fail to affect both approach and avoidance learning in the PSS task (Frank et al., 2007a) despite their clinical (Malhotra et al., 2002) and preclinical (Scheggia et al., 2013) linkage to cognitive flexibility. However, the PSS task measures approach and avoidance strategies in discrimination learning and inferences regarding its relevance for stimulus perseveration and learned non-reward in later reversal learning (Frank and O’Reilly, 2006) remain tentative. Moreover, the PSS task has generally been used to assess global pharmacological and genetic effects on discrimination learning. Yet the data from these studies are typically interpreted in the very detailed contexts of striatal D_1_R and D_2_R signalling. To enable conclusions regarding circuit-specific effects on stimulus perseverance and learned non-reward in reversal learning, such as the roles of D_1_R vs. D_2_R signalling, a preclinical paradigm suitable for non-human animals is required.

Finally, there are obvious methodological differences in the discussed studies that may account for inconsistencies observed in the literature. This includes the use of reversal learning assays underpinned by either Pavlovian learning or instrumental responding. Rodent reversal assays can also depend on instrumental responding with a strong spatial component as opposed to non-human primate and human tasks in which a wide array of visual discriminative stimuli are employed. The further development of touchscreen tasks in rodents provides one solution to this dilemma (Horner et al., 2013, Mar et al., 2013) though there has to be a residual concern about the ecological validity of this approach given the extended training that is necessary. Notably, task differences also appear to affect the main association guiding responding in discrimination and reversal learning. Responding can be primarily guided by the rewarded association (Nilsson et al., 2013, Robinson et al., 2010b, Tait and Brown, 2007, Waltz et al., 2007), the non-rewarded association (Jocham et al., 2011, Nilsson et al., 2012) or equally dependent on rewarded and non-rewarded associations (Clarke et al., 2007, Cools et al., 2008). Thus, slight protocol differences and species are likely to affect the relative guidance of approach and avoidance strategies.

In sum, investigations of neural substrates underpinning reinforced and non-reinforced associations in discrimination and reversal learning have used a wide variety of disparate paradigms, which is likely to influence the relative roles of approach and avoidance strategies and the effect of experimental manipulations. Nevertheless, the data show that perseverance and learned non-reward can be dissociable on anatomical, pharmacological, genetic and pathological levels, and that both perseverance and learned non-reward can contribute to the effect of an experimental manipulation on reversal learning performance.

## 6 Conclusions

Despite clear differences in symptom profiles across pathologies, most human psychiatric patients and animal disease models show relatively similar impairments on standard tests of cognitive flexibility. Current reversal learning assays may be rather crude measures of cognitive functioning that give few indications of possible pathology-specific deficits and therapeutic approaches. The Wisconsin Card Sorting Task (WCST) was recognised as a crude measure of cognitive flexibility and the task was successfully modified into a series of tests assessing the separate cognitive mechanisms required for attentional set-shifting in the more refined CANTAB ID/ED-task. Separate tests of perseverance and learned non-reward may have similar potential in delineating the neural basis of flexible associations with reward and non-reward in reversal learning.

Although manipulations of cognitive flexibility have traditionally been interpreted as manipulations of perseverance, the effects of learned non-reward and learned irrelevance must also be considered. This suggestion is supported both by empirical data and theoretical considerations from learning theory indicating that irrelevant or non-rewarded associations can be a major determinant of choice-behaviour in discrimination learning, reversal learning and attentional set-shifting tasks. Further work suggests that impairments of cognitive flexibility in different psychopathologies have different underlying behavioural mechanisms, and that the cognitive components of perseverance, learned non-reward, and learned irrelevance may be anatomically and neurochemically dissociable. This has important implications for the current understanding of reversal learning and attentional set-shifting and also has the potential to increase the validity of preclinical models of cognitive inflexibility.

Thus the resolution of reversal learning into separate tests of perseverance and non-reward will increase preclinical validity by clarifying species and task related differences in cognitive flexibility. A preclinical reversal learning task where choice-behaviour primarily is guided by associations of stimuli with non-reward is likely to have limited validity if choice behaviour in the clinical context is primarily guided by associations of stimuli with reward. Conversely, the demonstration that animal and human subjects solve analogous tasks using similar cognitive strategies by assigning comparable attention to cues of positive and negative association with reward would increase the validity of the task.

Thus assays of cognitive flexibility should be designed to separate effects of perseverance, learned non-reward and learned irrelevance. Dissociating these cognitive components into separate tests will enhance the validity of preclinical assays of cognitive flexibility and enhance the potential contribution of preclinical findings to improved mental health.

## Figures and Tables

**Table 1:** Observations of cognitive inflexibility in psychiatric disorders in reversal learning and attentional set-shifting tasks.

Underlying condition	Deficit observed in	Reference
Parkinson's disease	Spatial reversal learning	Freedman and Oscar-Berman (1989)
CANTAB ED perseverance/learned irrelevance probes	Owen et al. (1993)
CANTAB ED learned irrelevance probe	Slabosz et al. (2006)
Probabilistic visual reversal learning	Cools et al. (2001)
CANTAB set-shifting and reversal learning	Downes et al. (1989)

Alzheimer's disease	Spatial reversal learning	Freedman and Oscar-Berman (1989)
Object reversal learning	Freedman and Oscar-Berman (1989)

OCD	Probabilistic visual reversal learning	Remijnse et al. (2006)
CANTAB set-shifting	Chamberlain et al. (2006)
CANTAB set-shifting and Go/No-go reversal learning	Watkins et al. (2005)

Schizophrenia	CANTAB ED perseverance probe	Elliott et al., 1995, Elliott et al., 1995
CANTAB set-shifting and reversal learning	Ceaser et al. (2008), Jazbec et al. (2007), Leeson et al. (2009), Murray et al. (2008), Pantelis et al. (1999)

Autism	Spatial reversal learning	Coldren and Halloran (2003)
Probabilistic reversal learning	D’Cruz et al. (2013)
CANTAB set-shifting and reversal learning	Hughes et al. (1994), Ozonoff et al. (2004)
CANTAB set-shifting	Ozonoff et al. (2000)

Unipolar depression	Probabilistic visual reversal learning	Reischies (1999)
WCST	Martínez-Arán et al. (2004), Merriam et al. (1999)
CANTAB set-shifting and reversal learning	Taylor Tavares et al. (2007)

Bipolar depression	CANTAB set-shifting and reversal learning	Clark et al. (2001), McKirdy et al. (2009)
Probabilistic visual reversal learning	Gorrindo et al. (2005)
Go/No-go reversal learning	Holmes et al. (2008), Murphy et al. (1999)
Attentional set-shifting	Clark et al. (2005)
CANTAB set-shifting	Clark et al. (2002)
CANTAB reversal learning	Dickstein et al. (2004)
WCST	Martínez-Arán et al. (2004)

Huntington's disease	CANTAB set-shifting and reversal learning	Lange et al. (1995), Lawrence et al. (1996)
CANTAB ED perseverance probe	Lawrence et al. (1999)
Object reversal learning	Oscar-Berman and Zola-Morgan (1980)
CANTAB set-shifting	Lawrence et al. (1998)

ADHD	Go/No-go reversal learning	Itami and Uno (2002)
WCST	Reeve and Schandler (2001)
CANTAB set-shifting and reversal	Gau and Shang (2010), Kempton et al. (1999)

Cocaine abuse	Probabilistic reversal learning	Ersche et al. (2008)

*Note*. This table is not meant as an exhaustive list allowing comparisons between brain functioning and cognitive flexibility. Nor is it meant to stress the importance of cognitive flexibility impairments by ubiquity. Rather, the table draws attention to the non-selectivity of prevalent tasks of cognitive flexibility in discriminating between patient populations.

**Table 2:** Stimuli-reinforcement correlations in two-stage discrimination and reversal paradigms.

	Conditioning phase		Test phase	
Phenomenon	Stimulus A	Stimulus B	Stimulus A	Stimulus B
Reversal learning	+1.0	−1.0	−1.0	+1.0
Learned non-reward		−1.0		+1.0
Attentional set-shifting	+1.0	+0.5	+0.5	+1.0
Learned irrelevance		+0.5		+1.0
Learned avoidance	1.0†	0	0	0
Latent inhibition	0	None	1.0	None

In learned non-reward, a stimulus initially negatively correlated with reinforcement becomes positively correlated with reinforcement. In learned irrelevance, a stimulus initially non-correlated with reinforcement becomes correlated with reinforcement. In a typical learned avoidance tasks, a stimulus initially correlated with reinforcement becomes neutral. In an appetitive two-stage latent inhibition, an initially neutral stimulus becomes correlated with reinforcement.

† Aversive.

**Table 3:** Example of tests assessing the role of perseverance and learned non-reward in reversal learning through CS+ or CS− pre-exposure.

Test	CS+	CS−
Discrimination learning		
Perseverance test		
Single-stimulus pre-exposure	–	
Test-phase		
Learned non-reward test		
Single-stimulus pre-exposure		–
Test-phase		

To test the relative influence of perseverance or learned non-reward in reversal learning, subjects receive forced-choice pre-exposure trials to the reversed contingencies of either the previous CS+ or the previous CS− prior to the two-choice reversal challenge. If response behaviour in reversal learning primarily is guided by perseverance, pre-exposure to the reversed contingency of the previous CS+ should facilitate performance relative to pre-exposure to the reversed contingency of the previous CS−. If response behaviour in reversal learning primarily is guided by learned non-reward, pre-exposure to the reversed contingency of the previous CS− should facilitate performance relative to pre-exposure to the reversed contingency of the previous CS+.

**Table 4:** Example of tests assessing the role of reinforcement and non-reinforcement in discrimination and reversal learning by varying or replacing the CS+ or CS−.

Stage	Test	CS+	CS−
Discrimination learning	Reinforcement		
Non-reinforcement		
Reversal learning	Initial discrimination learning		
Perseverance		
Learned non-reward		

To assess the role of rewarded associations in discrimination learning, the CS+ is kept constant while the CS− varies across trials. Here, the only stimulus reliably predicting reward across trials is the rewarded stimulus. To assess the role of non-reinforcement in discrimination learning, the CS− is kept constant while the CS+ varies across trials. Here, the only stimulus reliably predicting reward across trials is the CS−. To assess the role of perseverance in reversal learning, the previous CS+ becomes CS−, while a novel CS+ replaces the previous CS. In this test, established non-reinforced association cannot guide responding as the previous CS− has been removed. To assess the role of learned non-reward in reversal learning, the previous CS+ becomes CS−, while a novel CS+ replaces the previous CS−. In this test, established associations of reward cannot guide responding as the previous CS+ has been removed (adapted from Clarke et al., 2007).

**Table 5:** Example of test assessing the role of perseverance using a 3-stimulus simultaneous discrimination and reversal paradigm.

Stage	CS+	CS−	CS−
Discrimination learning			
Reversal learning			

In discrimination learning, one stimulus is rewarded while two stimuli are non-rewarded. In the reversal test, the previous CS+ stimulus becomes a CS− and a previous CS− becomes the CS+. The second CS− remains non-rewarded in both discrimination and reversal learning. In this paradigm, responses to the previous CS+ are treated as perseverative while responses to the constant CS− controls for non-perseverative errors that occur when the subject is searching for an alternative response strategy. The positions of the stimuli vary pseudorandomly across trials (Jentsch et al., 2002).

**Table 6:** Example of tests separately assessing the role of perseverance and learned irrelevance in attentional set-shifting.

Stage	Stimuli		Relevant	Irrelevant	Correct stimulus
IDR			Shape	Lines	
Perseverance test			Solidity	Shape	
Learned irrelevance test			Lines	Solidity	

In a perseverance test of attentional set-shifting, the relevant dimension of the intradimensional reversal stage (IDR) becomes irrelevant while the previously irrelevant dimension is replaced by a novel relevant dimension. In a learned irrelevance test of attentional set-shifting, the irrelevant dimension of the IDR stage becomes relevant while the previously relevant dimension is replaced by a novel irrelevant dimension (adapted from Owen et al., 1993).

**Table 7:** Assessing stimulus avoidance and stimulus approach strategies in two-choice discrimination learning using the probabilistic stimulus selection task.

	Stimuli	
	Stimulus A (reward contingency)	Stimulus B (reward contingency)
Discrimination learning		
Pair 1	ま (80%/20%)	み (20%/80%)
Pair 2	そ (70%/30%)	の (30%/70%)
Pair 3	ら (60%/40%)	わ (40%/60%)
Test approach learning		
Pair 1	ま	そ
Pair 2	ま	の
Pair 3	ま	ら
Pair 4	ま	わ
Test of ‘avoidance’ learning		
Pair 1	み	そ
Pair 2	み	の
Pair 3	み	ら
Pair 4	み	わ

The table depict the principles of the probabilistic stimulus selection task (Frank et al., 2004, Frank et al., 2007b) and is not representative of individual studies using the task. Subjects initially acquire six separate stimulus reward contingences using three two-choice discriminations. Once acquired, the stimuli from Pair 1 are paired with the stimuli from Pair 2 and Pair 3 to form eight novel two-choice discriminations. Four discriminations tap stimulus approach learning and involve the stimulus most predictive of reward, while another four discriminations tap avoidance learning and involve the stimulus most predictive of non-reward. No feedback is given during discrimination tests of avoidance and approach learning.

**Table 8:** Assessing stimulus perseverance and learned non-reward in visual touchscreen reversal learning in the rat using interleaved probe trials.

	Stimuli	
	Stimulus A (reward contingency)	Stimulus B (reward contingency)
Discrimination learning		
Pair 1		
Pair 2		
Pair 3		
Reversal learning		
Full reversal test		
Learned non-reward test		
Perseverance test		

Animals initially acquire three separate stimulus reward contingences using three two-choice discriminations. Responses to the CS+ are rewarded, responses to the CS− are non-rewarded, and responses to the CS_50/50_ are rewarded on 50% of the trials. In the subsequent reversal stage, the previous CS+ becomes CS− and the previous CS− becomes CS+ while the CS_50/50_ remains unchanged. In probe trials of full reversal learning, the CS+ is paired with the CS−. On every 5th trial, animals are presented with a probe trial of perseverance or learned non-reward. In probe trials of learned non-reward, the CS+ (previous CS−) is paired with the CS_50/50_. Impaired performance in this condition indicates that previous negative associations guide responding. In probe trials of stimulus perseverance, the CS− (previous CS+) is paired with the CS_50/50_. Impaired performance in this condition indicates that previous positive associations guide responding.
